# Mitochondrial Functional Changes Characterization in Young and Senescent Human Adipose Derived MSCs

**DOI:** 10.3389/fnagi.2016.00299

**Published:** 2016-12-15

**Authors:** Bernd R. Stab, Laura Martinez, Adriana Grismaldo, Alejandra Lerma, María L. Gutiérrez, Luis A. Barrera, Jhon J. Sutachan, Sonia L. Albarracín

**Affiliations:** ^1^Department of Nutrition and Biochemistry, School of Sciences, Pontificia Universidad JaverianaBogotá, Colombia; ^2^Institute for the Study of Inborn Errors of Metabolism, Faculty of Sciences, Pontificia Universidad JaverianaBogotá, Colombia

**Keywords:** adipose derived mesenchymal stromal cells, mitochondria, reactive oxygen species, senescence, fission and fusion

## Abstract

Mitochondria are highly dynamic organelles that in response to the cell's bio-energetic state continuously undergo structural remodeling fission and fusion processes. This mitochondrial dynamic activity has been implicated in cell cycle, autophagy, and age-related diseases. Adult tissue-derived mesenchymal stromal/stem cells present a therapeutic potential. However, to obtain an adequate mesenchymal stromal/stem cell number for clinical use, extensive *in vitro* expansion is required. Unfortunately, these cells undergo replicative senescence rapidly by mechanisms that are not well understood. Senescence has been associated with metabolic changes in the oxidative state of the cell, a process that has been also linked to mitochondrial fission and fusion events, suggesting an association between mitochondrial dynamics and senescence. In the present work, we studied the mitochondrial structural remodeling process of mesenchymal stromal/stem cells isolated from adipose tissue *in vitro* to determine if mitochondrial phenotypic changes were associated with mesenchymal stromal/stem cell senescence. For this purpose, mitochondrial dynamics and oxidative state of stromal/stem cell were compared between young and old cells. With increased cell passage, we observed a significant change in cell morphology that was associated with an increase in β-galactosidase activity. In addition, old cells (population doubling seven) also showed increased mitochondrial mass, augmented superoxide production, and decreased mitochondrial membrane potential. These changes in morphology were related to slightly levels increases in mitochondrial fusion proteins, Mitofusion 1 (MFN1), and Dynamin-related GTPase (OPA1). Collectively, our results showed that adipose tissue-derived MSCs at population doubling seven developed a senescent phenotype that was characterized by metabolic cell changes that can lead to mitochondrial fusion.

## Introduction

Mesenchymal stromal/stem cells (MSC) were described by Friedenstein almost 50 years ago as plastic adherent fibroblast-like cells (Friedenstein et al., 1968, 1970, 1976). Caplan in 1991 referred to MSC as cells with regenerative potential in tissues of mesenchymal origin such as bone, cartilage, muscle, ligament, tendon, adipose, and stroma, thus he coined the term “mesenchymal stem cell” (Caplan, 1991). Multilineage differentiation potential was demonstrated by Pittenger et al. identifying this population as adult stem cells with stable phenotype (Pittenger et al., 1999). MSCs are highly metabolically, with a vast array of molecules secreted into the extracellular matrix (ECM), in addition to cytokines (Keating, 2012). Therefore, for the past 15 years MSCs have become very popular because of their therapeutic potential in tissue regeneration and cancer treatment (Schipani and Kronenberg, 2009; Dong and Caplan, 2012; Droujinine et al., 2013). The number of clinical trials on MSCs has been rising since 2004, including phase I–IV clinical studies for myocardial infarction, graft versus host disease, diabetes, spinal cord injury, and others. However, numerous scientific issues remain to be resolved before the establishment of clinical standards and government regulations (Wei et al., 2013).

Most therapeutic protocols require *ex vivo* cell expansion, guaranteeing reproducible, cost-effective, and good manufacturing practices. Although state of the art protocols describing systems such as microcarrier-based stirred cultured system have been reported (Carmelo et al., 2014), less is known about MSC expansion and senescence. To describe the phenotype acquired by MSC population after sequential cell passaging, characterized by low proliferation, and loss of clonogenic and differentiation potential, some researchers have used proteomic analysis to understand molecular mechanisms underlying replicative senescence (Madeira et al., 2012). Others have described epigenetic modifications during *in vitro* culture, where cells acquire DNA methylation changes at specific genomic sites (Schellenberg et al., 2014). Furthermore, telomere length and telomerase activity has been a hallmark during MSC expansion protocols (Parsch et al., 2004). Indeed it can be used as a method to track cellular changes upon long term culture (Wagner et al., 2010).

In addition to telomere shortening and telomerase activity inducing senescence, free radical, and mitochondrial theory are notable theories on aging (Andreyev et al., 2005; Romano et al., 2010). Decreased mitochondrial function is critical in the aging process and has been associated with age-related disorders (Seo et al., 2010). Mitochondria have been described as the major producers of free radical and concurrently the principal target of free radical action (Harman, 1972). The mitochondrial free radical theory of aging proposes reactive oxygen species (ROS), produced as by-products during normal metabolism results in oxidative damage (Sanz and Stefanatos, 2008). In response to a cell's bio-energetic state mitochondria are constantly remodeled. This process is known as mitochondrial dynamics, and is an integral part of many cellular responses. These dynamics are characterized by fission and fusion events that allow mitochondrial changes in orientation, number, and/or size within the cell. These tightly regulated processes allow constant remodeling of mitochondria (Hoppins et al., 2007). Conditions such as hypoxia, stress, and aging have been reported to impact mitochondrial dynamics leading to cellular dysfunction. Recently, mitochondrial fusion has been shown to induce senescent-like phenotypes in human cell cultures (Lee et al., 2007).

Mitochondrial fusion has been proposed to occur through two independent mechanisms in mammalian cells by which the inner and outer membrane fuse separately (Griffin et al., 2006). Fusion is believed to impart functional protection for the mitochondria by allowing them to exchange contents that might alleviate damaged constituents and promote repair (Seo et al., 2010). There are currently three established fusion proteins, Mitofusion proteins 1/2 (Mfn1/2) and optic atrophy protein 1 (Opa1). Over expression of Mfn 1/2 has been shown to inhibit apoptosis and promote cell survival (Sugioka et al., 2004). Furthermore, Fzo1, a protein involved in mitochondrial fusion, inhibits apoptosis (Sugioka et al., 2004). Mitochondrial fission participates in mitosis. In addition, it is thought to contribute to and even regulate apoptosis, as well as participate in the removal of dysfunctional mitochondria via mitophagy (Seo et al., 2010). In humans there are currently two established fission proteins, dynamin-related protein 1 (Drp1) and fission 1 protein (Fis1).

Reports in the literature suggest mitochondrial dynamics might play a role in senescence and aging, and could induce or alleviate these phenotypes (Ziegler et al., 2015). We proposed to study the mitochondrial structural remodeling process during *in vitro* culture. We set out to characterize possible fission and fusion events to establish the relationship between mitochondrial dynamics and replicative senescence.

## Materials and methods

### MSC isolation and population doubling determination

Mesenchymal stromal/stem cells were isolated from processed lipoaspirate. Adipose tissue was collected after informed signed consent from females (*n* = 3) undergoing cosmetic surgery with the approval of the Bioethics Committee at the Pontificia Universidad Javeriana. Processed lipoaspirate MSCs were isolated and characterized as previously described (Gutiérrez et al., 2013). Cells were passaged after reaching 80% confluency. Cell Media was changed twice a week, and MSCs were harvested and passaged according to the standardized protocol and re-seeded at a density of 10^4^ cells/cm^2^. Population doubling (PD) was established as follows:

(1) $$\begin{array}{l} \text{PD}=\text{log}\left(\text{N}/\text{Ni}\right)\times 3.32 \end{array}$$

N = number of viable cells, Ni = initial seeded number of cells.

### MSCs characterization

Cells at passage one were trypsinized and 96 × 10^3^ cells were seeded in six well plates and incubated with complete media for 24 h. Adipogenesis and osteogenesis was evaluated with media supplemented with inducers according to Gutiérrez et al. (2013).

#### Senescent phenotype

Mesenchymal stromal/stem cells were serially passaged until they reached a state of senescence defined as the inability to achieve 80% confluency after feedings over a 4 week period, verified by the senescence-associated β-galactosidase assay.

### Senescence-associated β-galactosidase assay

After reaching 80% confluency cells were detached and re-seeded at a 3 × 10^4^/6 well plate and incubated at 37°C, 5% CO_2_ for 24 h. Histochemical detection of β-galactosidase expression at pH 6.0 was only present in senescent cells and not pre-senescent cells, evidenced by x-gal conversion into a blue stain. Beta-galactosidase activity was evaluated following manufacturer's instructions (Senescence, β-galactosidase kit, Millipore, Massachusetts, USA). Last, SA β-gal cell stain count was performed under light microscopy and percentage of positive cells was calculated.

### Mitochondrial dynamics characterization

#### Mitochondrial mass

MSCs were stained with MitoTracker Green FM (Invitrogen, Carlsbad CA, USA) to quantify functional mitochondrial mass. Briefly, 48 h prior to staining 1 × 10^4^ cells were seeded using 35 mm glass bottom culture dishes, containing 14 mm microwell (MatTek, MA, USA). Cells were incubated for 30 min with DMEM supplemented with 80 nM MitoTracker Green FM, followed by 5 μM CellTracker Red CMTPX solutions to stain the cytoplasm (Invitrogen), and Hoechst (Invitrogen) to define cell nucleus. Cells were observed *in vivo*, and 2D and 3D images were collected by confocal microscopy using an Olympus FV1000 with an excitation/emission range of 400/545 for MitoTracker Green FM and 577/602 nm for CellTracker Red CMTPX. Z-stack parameters were as follows: ~15 z-axis slices at ~0.50 μm intervals with a final Z-stack thickness of ~7.5 μm. Mitochondrial mass was determined by data obtained from confocal microscopy in voxel units.

#### Mitochondrial membrane potential

Briefly, cells were collected as described for β-galactosidase assay and cultured for 48 h. We performed the established protocol for Mitoprobe™ JC-1 Assay Kit for Flow Cytometry (Molecular Probes, Invitrogen USA). We added 200 μM JC-1 and incubated for 30 min at 37°C. To determine mitochondrial membrane potential flow cytometry analysis was performed in a Guava easyCyte Flow cytometer (EMD Millipore, Billerica MA USA) at 488 nm excitation and 530 and 580 nm emission range with bandpass emission filters. Non-cellular debris and dead cells were gated out based on the light-scattering properties in the Side- and Forward- scatter parameters, and approximately 10 × 10^3^ events from live cells were collected for each analysis. Exposure to 50 μM carbonyl-cyanide 3-chlorophenylhydrazone (CCCP) (Sigma Aldrich, St Louis MO USA) for 10 min was used as a control to set the threshold of fluorescence intensity for cells with intact mitochondrial membrane potentials. Results are presented as the percent of all cells with Mitoprobe fluorescence greater than the threshold set by CCCP.

#### Mitochondrial anion superoxide production

After reaching 80% confluency cells were detached and re-seeded at a 3 × 10^4^/6 well plate and incubated at 37°C, 5% CO_2_ for 48 h. MitoSOX Red mitochondrial superoxide indicator for live-cell imaging (Molecular Probes, Invitrogen) assay was performed as an indicator of mitochondrial superoxide production. As a positive control 50 μM rotenone (Sigma) was used for 1 h. As a negative control we used 1X PBS without marker. Analysis was performed in Guava cytometer at 510/580 excitation/emission ranges. Results are presented as arbitrary units of fluorescence.

### Fusion and fission protein identification

Cells were harvested as described above in the cell culture method and lysed with lysis buffer (50 mM Tris-HCl, pH 7.5, 0.1 M NaCl, 1 mM EDTA, 10 mM MgCl_2_, 1% Triton X-100, and protease inhibitor cocktail). Sample protein concentration was determined by Bradford assay (Pierce). Equivalent amounts of proteins (25 μg) were separated by SDS-PAGE, transferred to nitrocellulose membranes, and immunoblotted. The following antibodies: MFN1 (Abcam, MA USA), OPA1 (Abcam, MA USA), DRP (Abcam, MA USA), and FIS1 (Abcam, MA USA), were used to determine if fusion or fission processes at the selected time-points were taking place. Immunoblots were visualized by the enhanced chemiluminescence system (JPI Healthcare Co, Plainview NY USA), β-actin (Abcam, MA USA) was used as the control and ProSieve QuadColor Protein Marker, 4.6–300 kDa as a reference for protein identification (Lonza, Allendale NJ USA). Protein quantification was performed in ImageJ (NIH) by normalizing mitochondrial dynamic protein to β-actin. Difference is reported as arbitrary units.

## Results

### MSC characterization and senescent phenotype

**Figure 1:**
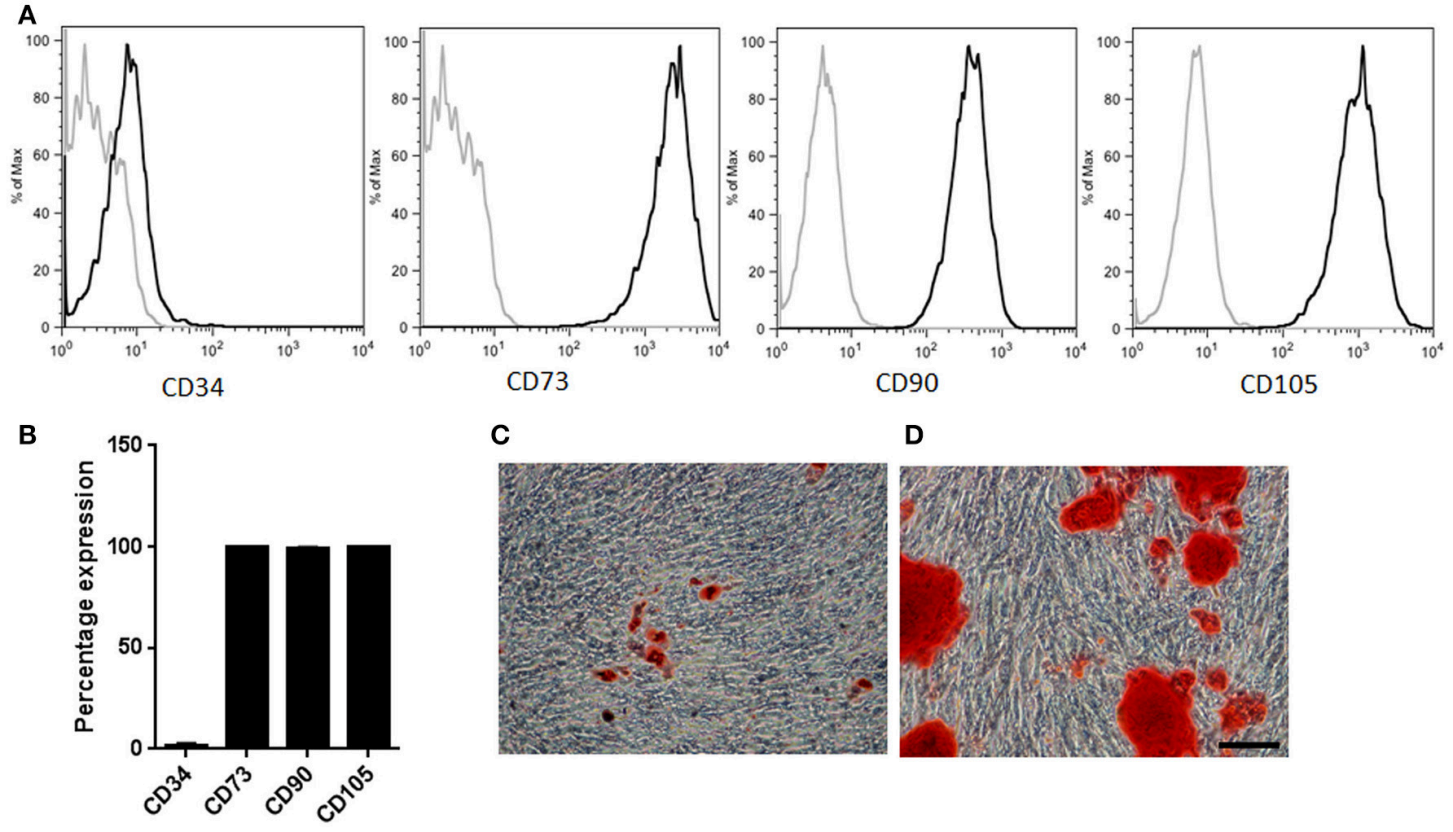
**MSCs characterization. (A)** Representative sample illustrating immunophenotypic profile. Antibodies against CD34, CD73, CD90, and CD105 (thick gray lines) with isotype control (thin gray lines). **(B)** Percentage expression corresponding to the average profile of all cell types (*n* = 3). **(C)** Representative sample of MSCs induced into adipogenic lineage differentiation. Neutral fat lipid droplets were detected by Oil red O stain **(D)** Representative sample of MSCs induced into osteogenic differentiation. Extracellular calcium deposits were determined by Alizarin Red stain. Scale bar 100 μm.

In order to characterize the effects of consecutive cell passaging on human MSCs proliferative capacity and mitochondrial metabolism, samples from three independently isolated healthy donors were cultured for seven consecutive passages (P1–P7) using DMEM supplemented with 10% FBS. Initial characterization at two population doublings (PD2) of these adipose derived-mesenchymal stromal/stem cells showed that these cells had high capacity of adherence to plastic, fibroblast-like morphology, and specific cell surface antigen expression of CD73, CD90, and CD105, and lack of CD34 expression (Figures 1A,B). Furthermore, adipose derived-mesenchymal stromal/stem were able to differentiate into adipogenic and osteogenic linages (Figures 1C,D) (Dominici et al., 2006; Gutiérrez et al., 2013).

**Figure 2:**
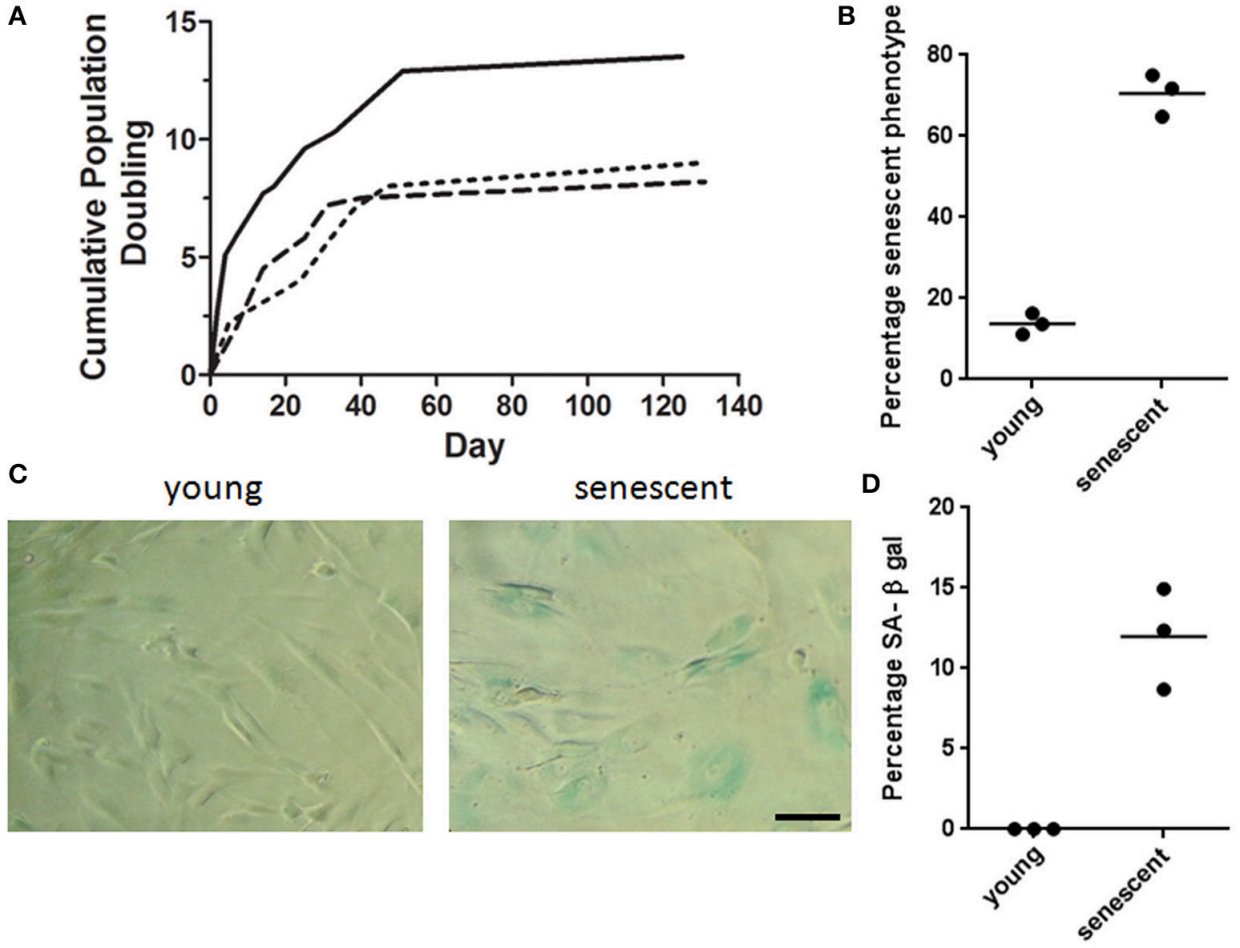
**MSCs senescent associated phenotype. (A)** Cumulative Population doubling for adipose derived MSCs. Adipose tissue derived MSCs were cultured for a total of 131 days. Cumulative PD were calculated at the end of every passage in relation to cell number of the first passage (*n* = 3). **(B)** Percentage senescent cells determined by cell count/field. Over 60% of MSCs at PD7 acquired a flattened and widened phenotype. **(C)** Representative brightfield images of young and senescent MSCs. Young cells are spindle shaped and slender in contrast to senescent cells with a widened and flat morphology. Cells presenting blue staining indicate the presence of β-galactosidase activity in senescent cells. Scale bar 100 μm. **(D)** Quantification of β-galactosidase positive cells for young (PD2) and senescent cells (PD7).

MSCs isolated from adipose tissue reached population doublings seven (PD7) after 130 days in culture and their growth was characterized by having a logarithmic proliferation rate during the first 40 days followed by a plateau phase in which MSCs were unable to achieve a 80% confluency over a 4 week period (Figure 2A). Upon light microscopy examination, significant changes in morphology were observed during consecutive passaging expansion. Comparison of MSCs morphology between PD 2 and 7 showed an increase in cell size, as well as a visible augmentation in cell granularity for PD7 cells. Namely, over 60% of MSCs at PD7 acquired a flattened and widened phenotype that was accompanied with a severe decrease in cell proliferation, suggesting that cells at PD7 were in a senescent state (Figure 2B). To further corroborate a senescent phenotype at PD7, we used the senescence-associated β-galactosidase stained assay (SA β-gal) with optimum activity at pH 6.0. In MSCs cultured up to PD7 we observed a marked increase in SA β-gal activity compared with PD2 (Figures 2C,D). Hence, we evidenced that MSCs cultured up to PD7 after 130 days in culture exhibited a senescent phenotype.

### Mitochondrial dynamics characterization

It is well established that MSCs can reach a senescent phenotype during *in vitro* proliferation and lose their differentiation potential, with alteration of their metabolic profile (Wagner et al., 2010; Geissler et al., 2015). Senescence has been associated with metabolic changes in the oxidative state of the cell, a process that has been linked to mitochondrial fusion and fission events (Mitra, 2013; Geissler et al., 2015). During the process of fusion and fission mitochondria can adjust their size, shape, and organization inside the cell (Westermann, 2010; Mitra, 2013). Significance of mitochondrial configuration changes has only recently begun to be understood, and might play a role in the regulation of senescence processes in MSCs. Therefore, we characterized mitochondrial changes for MSCs cultured at lower passage and higher passage number (PD2 vs. PD7) in adipose derived MSCs.

**Figure 3:**
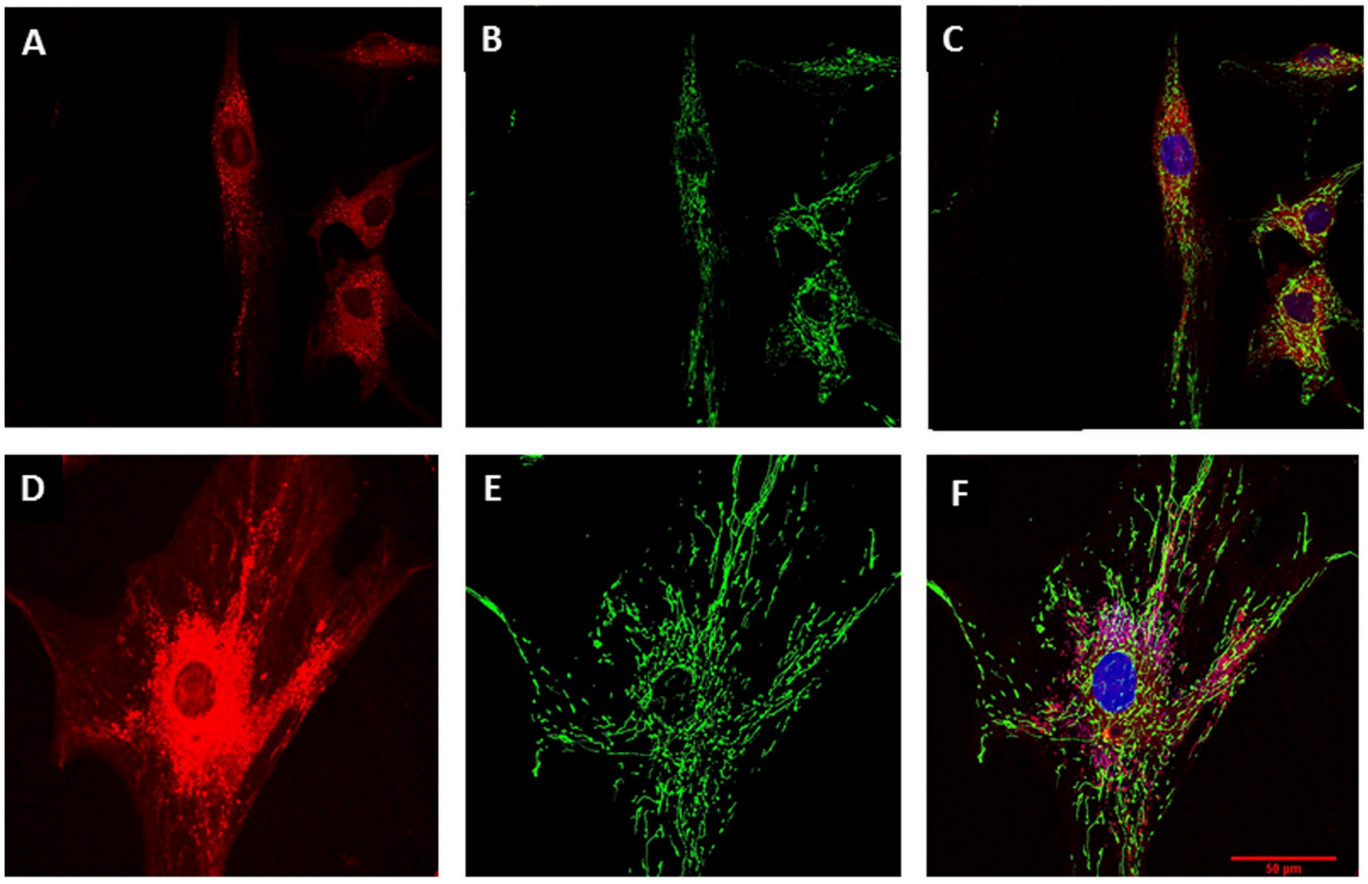
**Mitochondrial characterization**. MSC cell morphology and mitochondrial mass evaluation by confocal microscopy. **(A)** MSC at PD2 cytoplasm revealed by CellTracker red. **(B)** Mitochondrial stain with Mito Tracker green for MSCs at PD2. **(C)** Merge for **(A)** and **(C)** with nucleus stained with Hoechst (blue). **(D)** Larger cytoplasm in MSC at PD7 revealed by CellTracker red. **(E)** Mitochondrial stain with Mito Tracker green for MSCs at PD7. **(F)** Merge for **(A)** and **(C)** with nucleus stained with Hoechst (blue). Scale bar 50 μm for all images.

Morphological differences between young and senescent cells, as was described for bright-field microscopy, were also observed by using the CellTracker fluorescent dye (Figure 3). Digital rendering of Z-stack depicting the cell cytoplasm through CellTracker red evidenced young cell fibroblast morphology (Figure 3A) in contrast to senescent cells (Figure 3D), where an increased in cytoplasmic cell surface was observed. Likewise, morphological analysis of mitochondria at PD2 cells -using the mitochondrial marker MitoTracker green showed small tubular mitochondria forming a slightly interconnected network (Figures 3B,C). In contrast, PD7 cells had large tubular mitochondria forming an intricate network that was uniformly distributed in the cytoplasm (Figures 3E,F).

**Figure 4:**
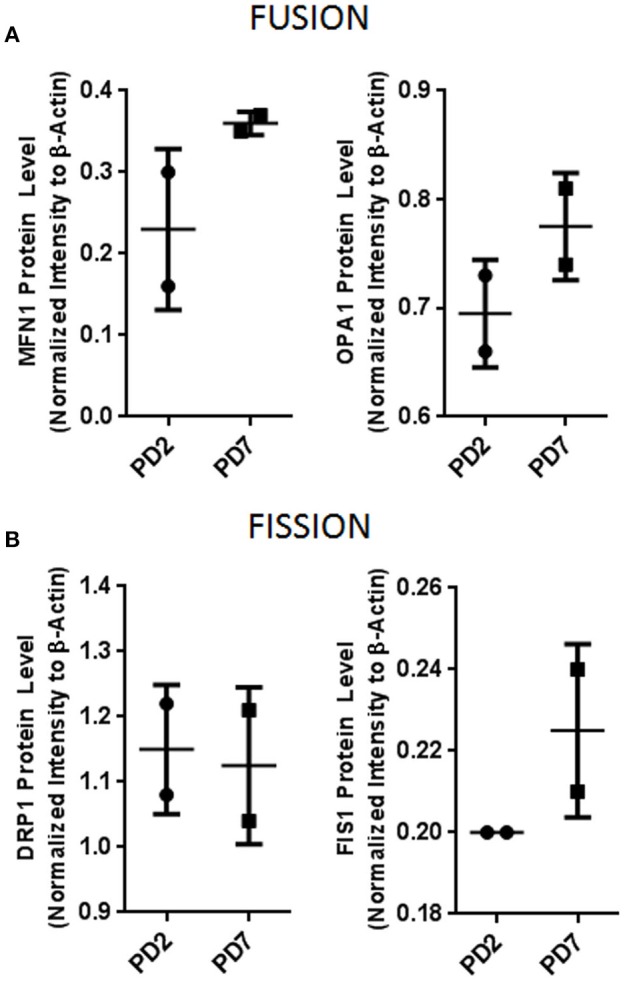
**Mitochondrial dynamics protein evaluation. (A)** Mitochondrial fusion proteins MFN1 and OPA1. **(B)** Mitochondrial fission proteins DRP1 and FIS1. Bar graphs depict protein expression as an arbitrary unit when protein of interest was normalized to β-actin. MFN1, Mitofusion1; OPA1, Optic atrophy 1; DRP1, Dynamin-1-like protein; FIS1, Mitochondrial fission 1.

To further corroborate if mitochondrial morphological changes were associated with mitochondrial volume, we quantified mitochondrial mass. Our results showed that PD7 MSCs had a larger mass (1286 ± 160) compared with PD2 (663.5 ± 104). These results suggest that increased adipose derived MSCs cell expansion lead to a senescent state characterized by an augmented mitochondrial network and mass. Mitochondrial dynamics fusion and fission are critical for organelle inheritance and maintenance of mitochondrial functions (Westermann, 2010). It has been described that increased mitochondrial mass could be associated with fusion processes. Therefore, we evaluated if changes in mitochondrial mass observed were due to changes in the levels of proteins involved in fusion and fission processes. Results showed a slight tendency for an increased level in fusion proteins GTPase, Mitofusion 1 (MFN1) and Dynamin-related GTPase OPA1 (Figure 4A), and in fission protein Fission 1 (FIS1) (Figure 4B). In contrast, fission protein DRP1 did not have important protein level difference between young and old populations. Collectively, our results suggest a tendency for fusion events in senescent cells, favoring the formation of mitochondrial networks. In response to mitochondrial stress fusion possibly protects the cell by maintaining a functional population of mitochondrial within the cell.

### Mitochondrial metabolic characterization

It has been shown that mitochondrial fusion is a mechanism used by the cell to dilute out mitochondrial dysfunction, respond to high energy demands, and maintain proper cell function. Since we observed mitochondrial mass changes in senescent MSCs associated with increased fusion, we then proceeded to evaluate mitochondria functionality by assaying mitochondrial membrane potential (Δψm) and ROS production (Zorov et al., 2014).

**Figure 5:**
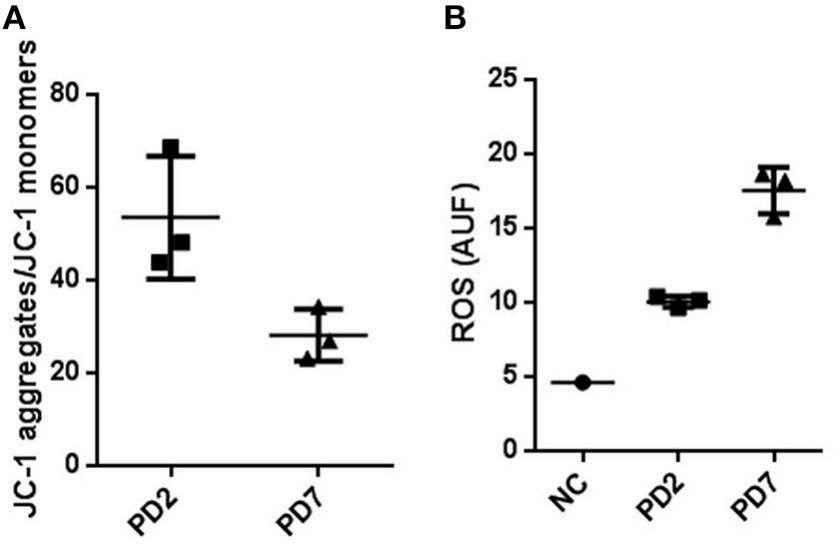
**Mitochondrial membrane potential and Reactive oxygen species production in young and senescent MSCs. (A)** FACS analysis to determine membrane potential changes by means of JC-1, a lipophilic cationic dye selectively entering into mitochondria. JC-1 accumulation in mitochondria, due to concentration-dependent formation of red fluorescent JC-1 aggregates was higher for MSCs cultured up to PD2 compared with PD7 MSCs. Results are presented as JC-1 aggregates/JC-1 monomer **(B)**. Reactive oxygen species production in young and senescent MSCs was compared by Mitosox Red fluorescent intensity production quantified by flow cytometry and expressed as arbitrary units fluorescence ROS (AUF). As a positive control rotenone at 50 μM was used.

Mitochondrial functionality by membrane potential was quantified by using the FACS JC-1 fluorescence dye. This cationic carbocyanine dye accumulates in the mitochondria in a low monomeric concentration yielding a green fluorescence when the membrane potential is low. On the other hand, it aggregates at high concentrations with a red fluorescence emission at high membrane potentials. Our results showed a 47% reduction in Δψm for cells at PD7 in comparison with cells at PD2 (Figure 5A). These results suggest that senescent MSCs have a diminished mitochondrial energetic capacity, which may lead to a lower activity in mitochondrial respiration and less ATP synthesis.

Subsequently, we characterized ROS production in young and senescent MSCs to evaluate the activity of mitochondrial respiration in these cells. Reactive oxygen species (ROS) are electron transporter chain (ETC) by-products of the cell's oxidative metabolism. A normal balance in ROS production and inactivation is mediated by different enzymatic systems and exogenous molecules capable of ROS detoxification to prevent oxidative stress damage (Sanz and Stefanatos, 2008). If mitochondria homeostasis is not sustained, higher ROS levels are produced. This in turn results in longer mitochondrial permeability transition pore (mPTP) openings releasing a ROS burst leading to mitochondria destruction. If these events are propagated from mitochondrion to mitochondrion, they might result in cell apoptosis (Zorov et al., 2014). Furthermore, reports have shown prolonged elongated mitochondria result in higher ROS production and lower mitochondrial respiration activity, resulting in cellular senescence (Yoon et al., 2007; Wagner et al., 2008).

Mitochondria superoxide production was evaluated by FACS analysis, where the reagent permeated live cells, selectively targeting mitochondria, and rapidly oxidizing to produce a highly fluorescent product. Our data determined anion superoxide production was 2.37 fold higher for senescent MSCs cultured up to PD7 compared with young MSCs (Figure 5B). Collectively, these results show that old MSCs have a decreased mitochondrial membrane potential, and increased ROS production, while young MSC have a conserved membrane mitochondrial potential and decreased ROS production. These results suggest that senescent MSC could have deficiencies in ATP production and an increase in oxidative stress. Old MSCs can compensate these mitochondrial changes with an augmentation in mass and interconnected mitochondrial network favored by the fusion process. Together, our results suggest possible mitochondrial functional changes that could be associated with senescence.

## Discussion

Mesenchymal stromal/stem cells have a great potential for therapeutic use; and human adipose tissue is an ideal autologous source of MSCs for diverse regenerative medicine and tissue engineering strategies (Caplan and Correa, 2011; Jin et al., 2013; Choudhery et al., 2014). Mesenchymal stem/stromal cells have been widely described in the literature for their therapeutic potential as anti-inflammatory, immuno-modulatory, and trophic support (Caplan and Correa, 2011; Choudhery et al., 2014). Most therapeutic protocols require *ex vivo* cell expansion to attain desired numbers, generally described as 1 × 10^6^/kg weight (Wagner et al., 2010). This requirement induces deficiency in cell proliferation capacity, loss of clonogenic potential, and impairment in differentiation potential (Madeira et al., 2012; Jin et al., 2013). Moreover, cells acquire DNA methylation changes at specific genomic sites brought about by epigenetic modifications during *in vitro* culture (Bentivegna et al., 2016). Therefore, MSCs proliferation and senescence are important issues to be considered for clinical safety and efficacy.

Our results demonstrate that for adipose tissue-derived MSCs a senescent phenotype was observed for cells cultured up to seven population doublings. This was evidenced by changes in cell morphology, decreased cell proliferation, and positive SA β-gal stain as previously described (Wagner et al., 2008). In addition to this characterization, we defined mitochondrial functional changes possibly related to replicative senescence to shed light on potential mitochondrial associated aging process. Senescent MSCs had an increased mitochondrial mass and strongly interconnected network that distributed uniformly in the cytoplasm, suggesting potentiation of fusion processes. Therefore, characteristic mitochondrial tubular shape was observed. In addition to slightly increased fusion associated proteins, MFN-1 and OPA1.

Mitochondrial dynamics are defined by the capacity of this organelle to continuously fuse or divide (Westermann, 2010). The balance between these two processes is responsible for mitochondria shape, distribution, inheritance, and functionality. If this delicate balance is lost, mitochondrial and cellular functions are affected as well (Detmer and Chan, 2007; Terman et al., 2010). Mitochondria can continually exchange contents through membrane fusion. This process controls organelle shape and is critically important for maintaining mitochondrial network function (Suen et al., 2008; Correia-Melo et al., 2016). We observed changes suggesting fusion events in senescent MSCs, based on a slightly higher MFN1 and OPA1protein levels. The most relevant proteins involved in mitochondrial fusion process are three GTPase dynamin-like proteins: Mitofusion 1 (MFN1) and 2 (MFN2), located in the outer mitochondrial membrane, and optic atrophy protein 1 (OPA1), in the inner membrane (Westermann, 2010). It is conceivable that under normal conditions outer membrane fusion is coordinated with inner membrane fusion. Even though we did not evaluate MFN2, our data evidenced slightly increased outer and inner mitochondrial membrane protein expression in aged MSCs. Under these conditions senescent MSCs would possibly use fusion processes to connect neighboring depolarized mitochondria and unite their contents to maintain membrane potential (Ikeda et al., 2015). Mitochondrial fusion has been reported to be beneficial for cardiomyocytes under stress conditions, since it consolidates the mitochondria's ability to supply energy (Ikeda et al., 2015). It is plausible this increased mitochondrial networking could be serving as a protective mechanism. It has been suggested mitochondrial fusion may protect neurons form excessive mitochondrial stress by maintaining a functional population (Ikeda et al., 2015).

In contrast, mitochondrial fission can segregate damaged mitochondria from intact ones, and the injured part of the mitochondria is phagocytized by mitophagy (Ikeda et al., 2015). Dynamin-related protein 1 (DRP1) is a master regulator of mitochondrial division (Westermann, 2010). Recruitment of DRP1 from the cytosol and assembly in the mitochondria depends partly on mitochondrial fission protein (FIS1). Both proteins are located on the external mitochondrial membrane. Our findings demonstrated younger cells expressed insignificant increased levels of DRP1, while senescent MSCs had a negligible increment in FIS1 expression.

Studies from in mouse embryonic fibroblasts (MEF) evidenced maintenance of mitochondrial morphological dynamics during cell stress can occur either by blocking fission (via dominant negative mutant-Drp1) or enhancing fusion (via Mfn1, Mfn2), suppressing mitochondrial injury and subsequent apoptosis. Furthermore, it has been reported Mfn1 and Mfn2 can maintain mitochondrial morphology during cell stress, and prevent mitochondrial outer membrane permeabilization and apoptosis (Brooks et al., 2011). In senescent MSCs, a hypothesis proposing fusion process enhancement mediated by Mfn1, could suppress apoptosis. Future studies associating intrinsic apoptosis signaling pathways would elucidate these mechanisms in senescent MSCs.

Among the principal pathways toward senescence reported in the literature associated with senescence are telomere shortening and ROS species production by mitochondria. To date to the best of our knowledge no study has addressed mitochondrial functional changes associated changes with replicative senescence in adipose derived MSCs. Mitochondria are involved in multiple anabolic and catabolic reactions, such as citric acid cycle and β-oxidation, in addition to their prominent role in metabolic energy production. Furthermore, they participate in developmental processes of aging (Westermann, 2010).

Our data show that old MSCs have a decreased mitochondrial membrane potential, and increased ROS production, while young MSC have a conserved membrane mitochondrial potential and decreased ROS production. These results suggest that senescent MSCs could have deficiencies in ATP production and an increase in oxidative stress.

The mitochondrial crisis can be described as a decrease in electron chain transport, diminished membrane potential Δψm, decreased oxidation, increased ROS production, and diminish mitochondrial function. Our data suggest a mitochondrial impairment could have occurred in senescent MSCs. Other authors have described for human fibroblast under oxidative stress during replicative senescence, ROS associated with increased mitochondrial mass (Lee et al., 2002). For the fibroblast study increase in mitochondrial mass was attributed to sublethal increased levels of oxidative stress. It is likely increased ROS production resulted in augmented mitochondrial mass, contributing to MSCs senescent phenotype. Furthermore, since Harman in 1956 proposed the free-radical theory of aging, where cumulative damage to molecules by ROS leads to irreversible cell damage and functional decline (Harman, 1956), this theory has also been extended including mutations and deletions in mitochondrial DNA. These accumulated age related mutations and deletions lead to impaired function of the respiratory chain and increase ROS production; generating a vicious cycle (Seo et al., 2010).

In conclusion, our study describes mitochondrial functional changes associated with adipose derived MSCs senescent phenotype after PD7. Increased MFN1 expression in PD7 MSCs, favoring fusion, could be suggestive of a protective mechanism against cells undergoing apoptotic events. Furthermore, metabolically MFN1 could potentiate membrane potential allowing the “senescent phenotype” MSC to adapt to stress. Our data suggest that the aging process could be associated with impaired mitochondrial function and energy metabolism in MSCs. However, further studies are required to define metabolic changes that accompany the mitochondrial crisis here described. Results derived from this study should be taken into account when considering MSC expansion for therapeutic use; as our data revealed mitochondrial functional changes associated with replicative senescence in cells with higher PDs.

**Figure 6:**
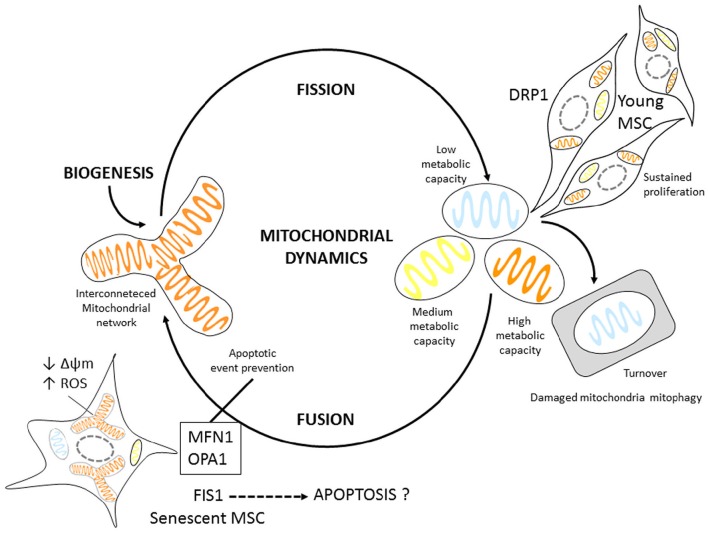
**Mitochondrial dynamic Model proposal for MSCs**. Young MSCs can readily undergo proliferation. Mitotic cells have slightly higher DRP1 expression, possibly regulating mitochondrial fission. Senescent MSCs display mitochondrial fusion. In addition they have larger mitochondrial volume; express proteins associated with fusion events most likely as an adaptation mechanism to energetic changes, such as increased ROS production, and decreased Δψm possibly to prevent apoptosis.

Our findings propose fusion and fission processes could be regulated under stress conditions for PD7 MSCs (Figure 6). Mitochondrial dynamics might be aiding in regulating mitochondrial ATP cellular levels and minimizing damaged mitochondrial DNA (mtDNA) accumulation during aging. We show fusion is important in senescent MSCs, perhaps to protect cells against oxidative stress and energy failure. During aging mitochondrial capability to generate ATP declines and mutations in mitochondrial and nuclear DNA accumulate (Stewart and Chinnery, 2015). However, to date the relationship between mitochondrial dynamics, and energy metabolism in young and senescent MSCs is still poorly understood and should be taken into account when considering MSCs for therapeutic applications.

## Ethics statement

Approved by the Bioethics Committee at the Pontificia Universidad Javeriana. Each donor carefully read the informed consent to donate a lipoaspirate sample during voluntary cosmetic surgery procedure. Any questions were answered by the researcher. The consent was signed by the donor with a witness.

## Author contribution

BS designed the work, acquired, and interpreted the data. LM, AG, and AL acquired and analyzed the data. MG, LB, JS, and SA interpreted the data, drafted and revised the work.

## Funding

This work was funded by Pontificia Universidad Javeriana-Vicerrectoría de Investigación, Projects 00004468 and 00007027, Mitochondrial fission and fusion: a new pathway toward senescence in Stem Cells.

### Conflict of interest statement

The authors declare that the research was conducted in the absence of any commercial or financial relationships that could be construed as a potential conflict of interest.
